# Bridging Organizations Drive Effective Governance Outcomes for Conservation of Indonesia’s Marine Systems

**DOI:** 10.1371/journal.pone.0147142

**Published:** 2016-01-21

**Authors:** Samantha M. Berdej, Derek R. Armitage

**Affiliations:** 1 Department of Geography and Environmental Management, Environmental Change and Governance Group, University of Waterloo, Waterloo, Ontario, Canada; 2 School of Environment, Resources and Sustainability, Environmental Change and Governance Group, University of Waterloo, Waterloo, Ontario, Canada; Tianjin University, CHINA

## Abstract

This study empirically investigates the influence of bridging organizations on governance outcomes for marine conservation in Indonesia. Conservation challenges require ways of governing that are collaborative and adaptive across boundaries, and where conservation actions are better coordinated, information flows improved, and knowledge better integrated and mobilized. We combine quantitative social network analysis and qualitative data to analyze bridging organizations and their networks, and to understand their contributions and constraints in two case studies in Bali, Indonesia. The analysis shows 1) bridging organizations help to navigate the ‘messiness’ inherent in conservation settings by compensating for sparse linkages, 2) the particular structure and function of bridging organizations influence governing processes (i.e., collaboration, knowledge sharing) and subsequent conservation outcomes, 3) ‘bridging’ is accomplished using different strategies and platforms for collaboration and social learning, and 4) bridging organizations enhance flexibility to adjust to changing marine conservation contexts and needs. Understanding the organizations that occupy bridging positions, and how they utilize their positionality in a governance network is emerging as an important determinant of successful conservation outcomes. Our findings contribute to a relatively new body of literature on bridging organizations in marine conservation contexts, and add needed empirical investigation into their value to governance and conservation in Coral Triangle nations and beyond.

## Introduction

A major challenge to effective conservation outcomes in the southeast Asia Coral Triangle (CT) is the ‘messiness’ of contemporary marine governance. People and groups bring different values, interests, perspectives, knowledge and power to conservation situations that span geographical and jurisdictional scales and levels (e.g., [1–5]). In Indonesia, decentralized governance and limited technical and financial capacity [4,6] further complicate definitions of effective conservation and efforts to achieve outcomes. Meaningful engagement is needed with actors and organizations, both government and nongovernment, to enhance coordination, improve information flows, and mobilize different sources of knowledge. These issues take us into the realm of governance, which we refer to here as the principles, rules, norms and institutions that guide public and private interactions to address challenges and create opportunities within society [7]. However, more collaborative and adaptive forms of governance that account for societal and ecosystem complexity are difficult to achieve [7–9]. The aim of this paper is to empirically investigate how bridging organizations contribute to better conservation outcomes by affecting key processes for adaptive marine governance in the CT context. We seek to examine in particular how regional and local-scale actors and actions may be better connected through the activities of bridging organizations, and how different forms of information, knowledge and resources may also be better exchanged.

Bridging organizations are defined here as entities that connect diverse actors or groups through some form of strategic bridging process [10]. Their relevance for collaboration and learning in adaptive governance contexts has been emphasized (e.g., [10–12]). One reason for an increased interest in such organizations is their utility as arenas for trust building, sense making, and conflict resolution where bridges are built, as for example, between science and other forms of knowledge (e.g., local knowledge), and between government and nongovernmental actors [13]. Recent evidence from different natural resource management settings shows that bridging organizations can add value to governing processes by providing platforms for coordination of actors and actions and shared learning, and by reducing the transaction costs of management (e.g., [12,14–16]). Still, few assessments of bridging organizations have been undertaken in the context of conservation governance generally, and in the CT region more specifically (although see [17] on Solomon Islands, [18] on Philippines). How such organizations affect, negatively or positively, the processes and conservation outcomes of governance in such situations requires further empirical examination.

Conservation challenges are inherently complex (e.g., diversity of stakeholders, scale). Adopting more collaborative and adaptive approaches to conservation governance is hypothesized to enhance successful outcomes in the CT and elsewhere (e.g., [6,18–21]). Such approaches are framed by three attributes: 1) interaction between diverse organizations and institutions that are linked with, and supported by, others at and across scales and levels [12,22], 2) continuous social learning where deliberative platforms for dialogue involve scientists, governments, resource users, and civil society to enable shared understanding, information transmission and integration of knowledge [23,24], and 3) social networks and bridging organizations as governance mechanisms to share responsibility, build trust and flexibility, and enhance collaboration and information flow (vis-à-vis attributes one and two) [11,13]. However, while such governance attributes have gained wide conceptual appeal, with some applications in CT contexts (e.g., [17,25–26]), their implementation in practice has been limited (e.g., [27]).

The CT region generally, and Bali Indonesia specifically, offers an instructive setting to examine the intersection of conservation, governance and the role of bridging organizations. The region is characterized by high marine biodiversity [28] and high dependence on coastal-marine systems for food security, livelihoods and culture [29]. Yet an array of threats from overfishing and other destructive fishing practices, land-based pollution, coastal development, and climate change are contributing to regional ecosystem decline [29–31]. The region falls under the policy umbrella of the Coral Triangle Initiative on Coral Reefs, Fisheries and Food Security (CTI-CFF), a multilateral partnership among six nations to jointly address marine resource issues ([32]–see below). The challenges of undertaking conservation in the CT are well documented, and include fragmented governance, complex institutional arrangements, misaligned scales of governing, competing objectives, and limited understanding and inclusion of social dimensions of resource use and conservation (e.g., [1,3–5,33–34]). Our analysis provides conservation managers, scientists and policy makers empirical insight on the value of bridging organizations as a key mechanism to grapple with ongoing conservation governance challenges in CT nations and other marine contexts.

We begin this paper with a brief outline of the research context, focusing on two study sites in Bali, Indonesia. The methods used for data collection are then described, and include questionnaires and social network analysis (SNA), semi-structured interviews, observation and literature review. This approach mixes quantitative and qualitative methods of data collection and analysis for a mutually informative research process. A mixed method approach is useful as a way to explore the structural and relational characteristics of bridging organizations from an ‘outsider’ perspective, along with attention to the meanings and outcomes of bridging from an ‘insider’ perspective. The results focus first on identifying and characterizing bridging organizations and their networks. Second, we show the attitudes and perceptions of respondents about bridging organizations in question, and their contributions to coastal-marine governing processes and conservation. The discussion explores opportunities and challenges for inclusion of bridging organizations in facilitating adaptive governance processes that can lead to better conservation outcomes. We offer conclusions to help nest these insights in broader conservation contexts, and point to future research directions/needs.

## Materials and Methods

### Study Sites: Conservation and Governance along the Balinese Coast

**Fig 1:**
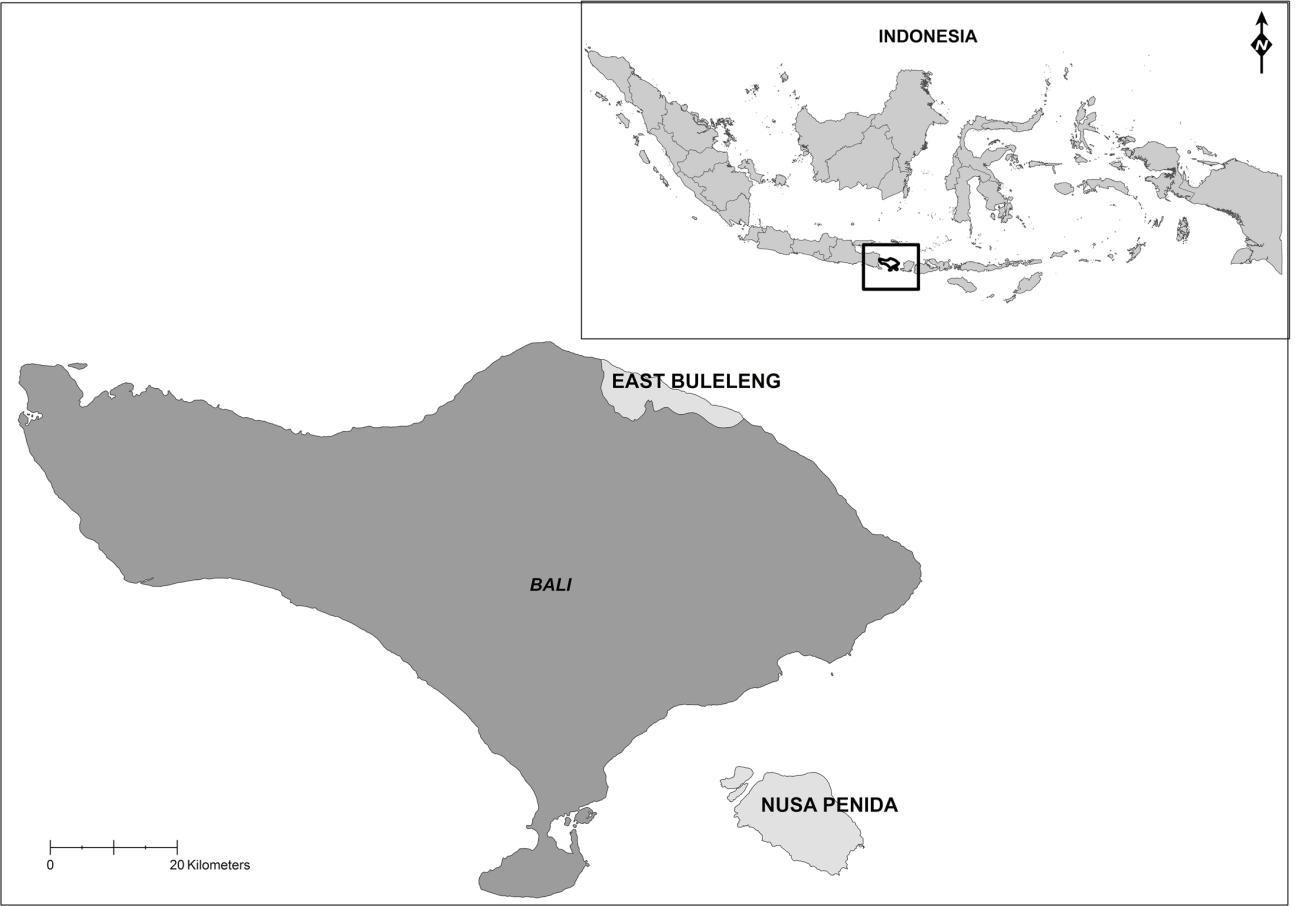
Map of Bali, Indonesia showing the two research locations: Nusa Penida MPA and East Buleleng Conservation Zone. Made with Natural Earth.

The Indonesian province of Bali is located in the westernmost end of the Lesser Sunda Islands (Fig 1) in the Coral Triangle (CT) region of southeast Asia, a global center of marine biodiversity and abundance [28]. The province supports close to 4 million inhabitants, the majority of which are intimately linked to the sea as a source of livelihoods, food security and culture. In 1999, a series of local autonomy laws transferred authority and responsibility to manage coastal and marine resources from the national level to sub-governments, granting local governments (regencies and city) almost absolute authority over the natural resources within four nautical miles of the coastal shoreline [35]. This shift has resulted in conflicts, confusion and questions within the Indonesian legal system about laws made at different levels of government (see [35]). A variety of government bodies (e.g., Ministry of Marine Affairs and Fisheries, Ministry of Forestry), local governments (provincial and regency) and others (e.g., NGOs, universities, community groups) help to manage the Balinese coast (see below). These management bodies are in addition to existing local traditional authorities (e.g., Adat) and customs (e.g., sasi, awig-awig), which vary by strength across different regions (see e.g. [36]). This customary law outlines rights, rules and sanctions associated with the interactions and management of natural resources in a given area. By management we refer to the operational decisions and practices in natural resource use that influence governance [8].

Increased pressure on marine resources in Bali and elsewhere in the CT has resulted in local, national and global initiatives to improve governance of coastal-marine ecosystems and conservation outcomes. The Coral Triangle Initiative on Coral Reefs, Fisheries and Food Security (CTI-CFF), as mentioned above, is one such initiative. The multilateral partnership among Indonesia, Philippines, Malaysia, Papua New Guinea, Solomon Islands and Timor Leste was adopted to address threats to coastal and marine environments, while also seeking to alleviate poverty and ensure food security in the region [32]. A Regional Plan of Action was collaboratively developed, which outlines core principles, goals and targeted actions for the next 10 years. The CTI-CFF aims to achieve these “…*through accelerated and collaborative action*, *taking into consideration multi-stakeholder participation*…” ([37], page 1). The Plan of Action is supported by a number of actors, including CT country governments, international NGOs, US and Australian governments, and the academic community.

Two study sites in Bali were chosen for their mixture of land and sea use activities, and the diverse social and environmental pressures they face: 1) Nusa Penida Marine Protected Area, south Bali, and 2) East Buleleng Conservation Zone, north Bali (Fig 1).

#### Nusa Penida Marine Protected Area, south Bali

Nusa Penida is an island chain southeast of the Balinese coast and is under the administration of the Klungkung Regency, Bali Province (Fig 1). Approximately 48,000 inhabitants are spread over three islands: Nusa Penida, Nusa Cenigan, and Nusa Lembongan. The MPA is host to highly diverse coral ecosystems and large charismatic species such as the mola mola (sunfish), manta rays, and sharks, and sees some 200,000 tourists per annum [38]. Still, marine areas are overexploited because of competing income-generating activities, including seaweed production, aquaculture, capture fisheries and marine tourism [39]. Other threats to biodiversity include pollution, sewage, destructive anchoring practices, coral mining, coastal development and climate change. The area was declared a national MPA in 2010 and was gazetted in 2014. The MPA is governed by the newly established Nusa Penida MPA Management Unit under the administration of the Ministry of Marine Affairs and Fisheries, Klungkung. The Unit consists of representatives from multiple agencies, and includes a joint patrol team and resource use monitoring experts. Other key groups include community associations, private sector diving associations and traditional bodies (*Adat*) who have localized regulations and codes of conduct–see Table 1.

**Table 1:** Typology of organizations in the Nusa Penida MPA & East Buleleng Conservation Zone.

Type	Scale	Description
Fishers’ association	local	Geographically-defined cooperatives of fishers
Ornamental fishers’ association	local	Geographically-defined cooperatives of fish collectors–East Buleleng only
Seaweed farmers’ association	local	Family or geographically-defined cooperatives of seaweed farmers–Nusa Penida only
Community-based organization	local	Organizations within communities defined by shared experience or concerns
Traditional authority	local–regency	Customary territorial authorities
Monitoring & enforcement agency	local–national	Formal and informal regulatory and monitoring bodies
Government agency	local–national	Government bodies with interest or authority over resources or geographic territories
Non-government organization	local–int’l	Non-profit organizations defined by common interests and organized around specific issues
Private enterprise	local–int’l	Private businesses or operators associated with the tourism industry
Funding organization	local–int’l	Donor or funding body

#### East Buleleng Conservation Zone, north Bali

The Tejakula sub-district is located in north Bali and is under the administration of the Buleleng Regency, Bali Province (Fig 1). In 2013 the sub-district was home to approximately 54,000 peoples in ten villages [40], of which we focus on four: Bondalem, Tejakula, Les, and Penuktukan. One of the poorest regions in Bali, marine-based livelihoods here include pelagic fisheries, the marine aquarium trade and tourism. North Bali has a tumultuous history associated with destructive fishing practices involving cyanide and dynamite, but has since reformed to be a leading exporter of ornamental fish (see [41]). The area has been identified as a future location for the development of marine tourism. Ongoing marine pressures include plastic waste, illegal fishing and fish collection, destructive fish practices and coastal development. Ecosystems in this region are governed by the Regency through marine and fisheries legislation, but also by community associations who have localized regulations and codes of conduct–see Table 1. Here it is common for community members to hold membership in multiple associations simultaneously. In addition to village-level Marine Management Areas (MMA) that were started in 2008–2009, the district as a whole was recently declared an MPA that is divided into three units. The East Buleleng MPA unit covers the waters in front of all nine villages in the sub-district Tejakula and is currently the focus of zoning and planning processes.

### Ethics Statement

The research project was approved by the Office of Research Ethics of the University of Waterloo (Ethics Approval Number 17930). A permit was secured to conduct research in Bali, Indonesia (permit number 393/SIP/FRP/X/2013). Verbal consent was obtained from participants prior to conducting questionnaires and interviews. During the consent process, an information sheet detailing the purpose of research and how data would be utilized was read and/or translated verbally to participants. This also specified their rights to withdraw participation from the research at any time. Individual names were not recorded, however, participants were given the choice to be identified by organizational affiliation or anonymously. The use of verbal consent was approved by the ethics committee prior to undertaking field activities.

### Methods

Data collection occurred in two study sites over an eight-month period between 2013–2014, with a follow-up verification phase in January-February 2015. Research methods included: 1) semi-structured questionnaires to collect network data (n = 43 Nusa Penida, n = 48 East Buleleng) and 2) in-depth interviews to collect respondent attitudes and perceptions (n = 53 Nusa Penida, n = 54 East Buleleng) with a broad range of actors in each site (e.g., resource users, government agencies, NGOs, community groups, traditional authorities, private sector representatives)–Table 1. Other methods included observation of public MPA planning meetings to gather information on coordinated activities (two per site), and a literature review of related documentation (e.g., annual reports, internal documents, policy briefs, newspaper articles, etc.).

Participants were identified using a non-probabilistic snowball sampling technique [42] where individuals nominate subsequent participants, starting with key organizations in each of the networks. Snowball sampling is a common technique used in qualitative research [43] and is helpful to local ‘hidden populations’ or key individuals that otherwise would not have been known. In addition, snowball sampling is useful to obtain research or knowledge about the social network connecting actors or groups. We chose this technique given the diversity of stakeholders included in our study that made defining an adequate sampling frame difficult. Participant sampling continued until the point of data saturation was reached where no new information or insights were yielded.

Social network analysis (SNA) [44] was used to map, describe and analyze the patterns of how organizations interact with a particular focus on application in conservation settings (e.g., [45]). Network data was gathered via questionnaire by asking respondents three separate questions about the relationships among their organization and others according to different network configurations: collaboration, knowledge-exchange and funding or resource sharing (see Table 2). Each configuration represents a different process for governance. The questionnaire focused on organizations, not individuals, and used prompted recall-based elicitation of network data. Using questionnaire responses we assigned organizations to groups based on their type (see Table 1).

**Table 2:** Different types of social network configurations examined, and the chosen questions used to elicit information.

Configuration name	Type of network	Question posed
Collaboration configuration	Participation in shared actions or interactions, strategies, technical partnerships, etc.	Q1. With whom do you most often collaborate on marine projects or issues? These issues may include management plans, fieldwork, joint campaigns, etc.
Knowledge-exchange configuration	Exchange of information or knowledge about coastal-marine environment and/or resources	Q2. With whom do you most often share information or knowledge about the marine environment? This knowledge may include scientific data, history, advice, perspectives, concerns, etc.
Funding or resource-sharing configuration	Sharing of financial or non-financial resources such as equipment, office space, machinery, etc.	Q3. With whom do you receive/share/give funding or other resources? Other resources may include lending equipment, office space, boats, etc.

SNA focused on two calculated measures of centrality: 1) betweenness centrality and 2) in-degree centrality [sensu 44,48]. Certain structural and relational characteristics are linked in theory to governance processes and outcomes (e.g., [24,46–47]), including those associated with collaboration and learning in bridging organizations (e.g., [10]). Betweenness calculates the number of shortest paths that run through an organization, indicating power and importance for connecting others in the network who were not otherwise connected [48]. The more ‘in between’ an organization might be, the better able that organization is to access and diffuse different types of knowledge and information among others in the network [47]. Importantly, there can be multiple organizations in a network with high betweenness centrality scores at the same time. Betweenness is a useful measure to consider because it aligns with how many scholars structurally conceive the concept of bridging organization (e.g., [10,11]). In contrast, in-degree is an indicator of the popularity or prestige of an organization in the network, and measures the number of connections an organization receives from other organizations [44]. Because they have many connections, these organizations are considered to be ‘hubs’, and are better able to exert influence over others in the network. Taken together, analysis of these measures is a first step to identify and characterize bridging organizations in a network.

Key individuals (n = 107) were interviewed in-depth to assess, among other things, their perception and attitude of how bridging organizations impact social processes and network members with reference to key governance processes hypothesized to lead to successful conservation outcomes (e.g., participation, coordination, collaboration, cross-level, deliberation, learning, knowledge-exchange). Interviews lasted 30 to 90 minutes. Questions focused on the contributions of bridging organizations to coastal-marine governance and conservation with regards to: a) collaborative and knowledge-exchange (learning) processes in the network as a whole, and b) changes within individual organizations as a result of direct bridging organization intervention. Lastly, respondents were asked to reflect on the constraints and barriers to establish or strengthen new relationships in each of the networks. Results from interviews have been corroborated with other sources of information (e.g., annual report, newspaper articles), as well as shorter follow-up verification interviews conducted January-February 2015.

## Results

Results are presented here in two parts. First, we synthesize the outcomes of the SNA to map and characterize the network in each case and to identify bridging organizations using measures of centrality. We review what organizations are involved in collaborative, knowledge-exchange and funding or resource-sharing relationships, what organizations connect or facilitate these relationships, and what organizations reside in positions of influence. In the second part we analyze respondent perceptions and attitudes of bridging organizations to distinguish functionality and their effects on social processes and organizations in the network with regard to coastal-marine governance. We draw on examples from the field to demonstrate their implications for conservation outcomes.

### Network Structure & Identifying Central Organizations

#### Nusa Penida MPA

**Fig 2:**
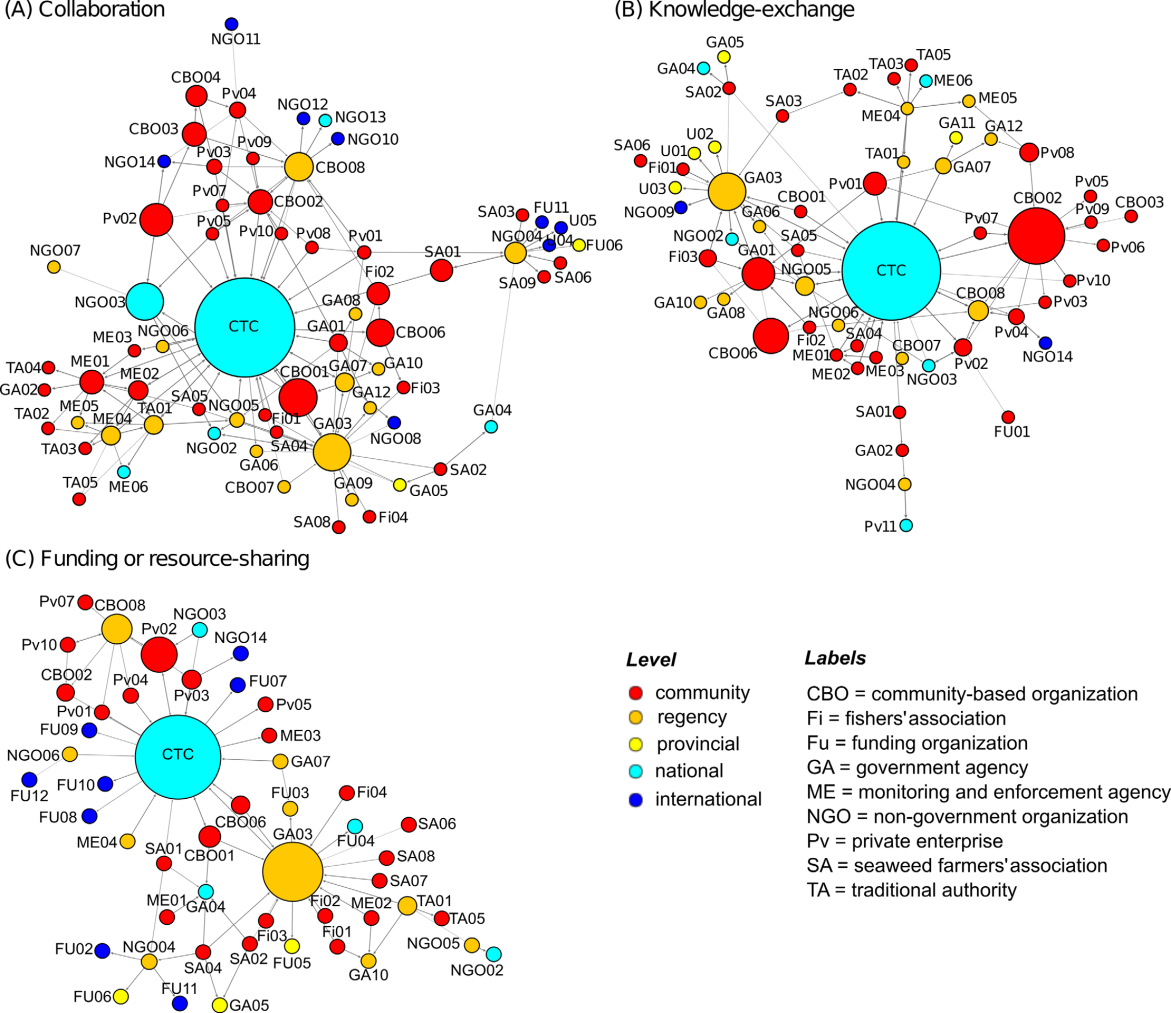
Network maps of the Nusa Penida MPA network. Network maps illustrate relationships (represented by lines) between organizations (represented by circles) associated with the network. The size of the circle indicates its betweenness centrality (bigger circles = higher betweenness) and the colour of each circle indicates its level. Betweenness measures based on: (A) collaborative relations (n = 67), (B) knowledge-exchange relations (n = 59) and (C) funding or resource-sharing relations (n = 50). Labels are composed of the type of organization, and a unique number to distinguish them from others in the group.

Respondents identified 86 organizations in the Nusa Penida MPA network, representing various sectors of society and divergent interests (see Table 1). These are organizations that could affect, or be affected, by marine resource governance and conservation decisions to varying extents. Of these, the collaborative configuration registered 67 organizations connected by 141 relations, the knowledge-exchange configuration registered 59 organizations connected by 100 relations, and the funding configuration registered 50 organizations connected by 72 relations. The network maps in Fig 2 illustrate these findings as relational patterns of collaboration (panel A), knowledge-exchange (panel B) and funding or resource-sharing (panel C) (see also Table 2). The network was largely dominated by local organizations (shown in red, Fig 2A, 2B and 2C) and regency-level organizations (in orange). Analysis showed a low density of connections in all network configurations (i.e. few connections between organizations). Fig 2A and 2B highlight several distinguishable clusters where organizations are more closely connected to one another than the rest of the network.

An examination of betweenness indicated that in all three network configurations the Coral Triangle Center (CTC), a national NGO, held the maximum score for ‘bridging’ or connecting otherwise disconnected organizations (Table 3). In Fig 2A, 2B and 2C the sizes of the nodes are proportional to betweenness scores. As explained, because of its location ‘between’ others, it is implied that the CTC is a natural coordinator or broker of collaborations, and can control or influence the flow of information or resources within the network (e.g., [47,11]). Those organizations with the second highest betweenness rankings included two community-based organizations and a regency government agency. Even so, the overall scores between the first and second place organizations were significant–in one case the CTC’s betweenness score was more than three times greater than that of the organization with the second highest score. SNA data clearly demonstrates that the CTC plays the most central bridging role in the network and is thus of focus in this paper. Details of the betweenness scores for the top ten ranking organizations in the network are given in S1 Table.

**Table 3:** Betweenness and in-degree centrality measures of highest scoring organizations within the Nusa Penida and East Buleleng governance networks.

Configuration type	Nusa Penida		East Buleleng					
	Coral Triangle Center		Reef Check Indonesia		DKP Buleleng		LINI^b^	
	between	in-degree	between	in-degree	between.	in-degree	between	in-degree
**Collaboration**	1158.3	24	366.5	16	355.3	17	-	14
**Knowledge-exchange**	839.3	22	302.6	11	220.7	15	226.7	7
**Funding & resource sharing**	491.5	12^a^	77.2	5	94	14	-	-

^a^ This is the second highest in-degree measure in the network. The highest is attributed to the Ministry of Marine Affairs and Fisheries, Klungkung (in-degree = 13)

^b^ Only measures that ranked in the top three in the network were included here (i.e. LINI has a high betweenness measure for knowledge-sharing, but a medium to low betweenness measure for collaboration and funding)

When considering in-degree measures (Table 3), the highest scores in the collaboration and knowledge-exchange configurations were also attributed to the CTC (i.e. it had the largest number of connections with others in the network). The NGO connected with 20 community organizations, nine district, one national and four international organizations, represented from a variety of sectors. Others with a relatively high number of connections included the Ministry Marine Affairs and Fisheries, Klungkung (DKP-K) with regards to funding and resource sharing. Nevertheless, a high level of betweenness and high in-degree suggest that CTC is an important bridging organization in the Nusa Penida MPA network.

#### East Buleleng Conservation Zone

**Fig 3:**
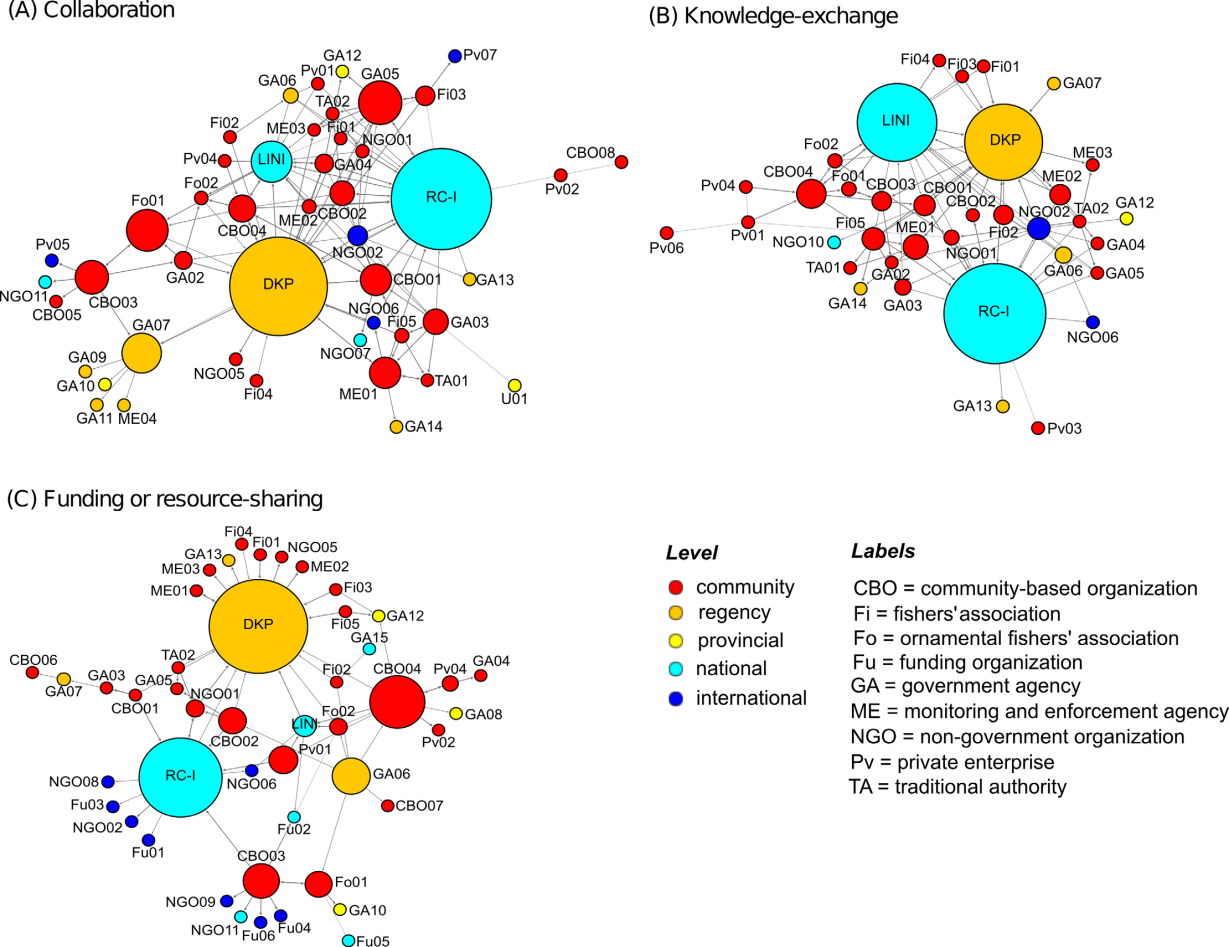
Network maps of the East Buleleng Conservation Zone. Network maps illustrate relationships (represented by lines) between organizations (represented by circles) associated with the network. The size of the circle indicates its betweenness centrality (bigger circles = higher betweenness) and the colour of each circle indicates its level. Betweenness measures based on: (A) collaborative relations (n = 46), (B) knowledge-exchange relations (n = 36), and (C) funding or resource-sharing relations (n = 46). Labels are composed of the type of organization, and a unique number to distinguish them from others in the group.

The respondents identified 62 organizations in the East Buleleng Conservation Network from differing sectors and scales (overview in Table 1). Similar to the Nusa Penida, these are organizations that could affect, or be affected, by marine resource governance and conservation decisions to varying extents. The collaborative configuration registered 46 organizations connected by 137 relations, the knowledge-exchange configuration 36 organizations connected by 91 relations, and the funding configuration 46 organizations connected by 69 relations. The network maps in Fig 3 illustrate these findings as relational patterns of collaboration (panel A), knowledge-exchange (panel B) and funding or resource-sharing (panel C) (see also Table 2). Local organizations (shown in red, Fig 3A, 3B and 3C) and international level organizations (in dark blue) constitute the two largest groups. Comparatively, organizations in the East Buleleng network are proportionally better connected to one another, but overall network cohesion is still low.

Three organizations from both the NGO and government community shared the highest ‘bridging’ scores in the East Buleleng Conservation network: 1) Reef Check Indonesia (RC-I), a national-level NGO, 2) the Ministry of Marine Affairs and Fisheries, Buleleng (DKP-B), a regency-level government agency, and 3) the Indonesian Nature Foundation (LINI), a national-level NGO. Collectively, these organizations were important to help coordinate or facilitate collaboration, influence the flow of information or knowledge, and/or influence the flow of funding or resources in the network, although not equally so (see Table 3). For instance, LINI helps to moderate information flow within and across the network, but is not especially involved in brokering relationships or affecting the flow of funding and resources. High betweenness scores were similar among the first and second place rankings of organizations (in the collaboration and funding configurations, respectively), and among the first, second and third rankings of organizations (in the knowledge-exchange configuration). Details of the betweenness scores for the top ten ranking organizations in the network are given in S2 Table.

Those with highest betweenness also tended to have high in-degree measures, meaning that while these organizations have a great many connections they also tended to form these connections across others that are more disconnected. DKP was linked to 20 community organizations, two district, two national and two international organizations; RC-I was connected with 15 community organizations, four district, one national and five international organizations; and LINI was connected with 16 community, two district, two national and two international organizations. All three organizations are also connected to one another, as referenced in the following sections. Taking both measures together, Table 3 helps to identify central actors that might be playing more active and influential roles in the network.

### Functionality of Bridging Organizations in Governance Networks

We complemented our SNA findings with in-depth interviews designed to assess the functionality and perceived impacts/influence of each bridging organization for coastal-marine governance, and their subsequent implications for conservation outcomes. Only those respondents who stated they had interacted with the bridging organization in question on one or more occasions over the last year are included in the results. Results from interviews were complemented with other secondary information sources. The main results are summarized in Table 4 and discussed in-depth in the sub-sections below. Results are presented by case and by bridging organization. A brief description and history of each organization is given, followed by respondent perceptions of the roles and contributions of bridging organizations, and their implications for conservation outcomes. Finally, we discuss the constraints and barriers for building new relationships in each case.

**Table 4:** Summary of findings on main bridging organizations in Nusa Penida MPA and East Buleleng Conservation Zone.

	Nusa Penida	East Buleleng		
	Coral Triangle Center	Reef Check Indonesia	DKP-Buleleng	LINI
**Type**	NGO (national)	NGO (national)	Government (regency)	NGO (national)
**Network community**	Three Nusa Islands	Buleleng Regency (focus Tejakula sub-district)	Buleleng Regency	Buleleng Regency (focus Les & Penuktukan villages)
**Focus of activity**	Establishing and implementing an MPA in Nusa Penida	Supporting community-led sustainable marine resource management	Actualizing management of fisheries and ensuring welfare of resource users in the Regency	Creating a sustainable marine ornamental fishery
**Roles and contributions**	Provide expertise on the est. of MPA plan	Liaise with gov’nt to facilitate local and sub-district MPAs	Coordinate sub-district MPA zoning plan and associated activities (e.g., face-to-face meetings)	Building local capabilities for ornamental fisheries by transferring skills and knowledge to local community
	Coordinate and empower collective MPA forums (e.g., working group, mgmt. unit)	Support and empower community-based institutions like MMAs by e.g., building capacity	Provide financial and in-kind support to marine resource user groups	Coordinate data collection and management, and distribute data sets to relevant parties
	Help to catalyze local institutions and forums for interaction	Coordinate with local community to conduct education and awareness programming	Facilitate legal grounds for conservation	
	Carry out educational programming and promote ‘learning sites’			
**Implications for conservation outcomes**	Balance of multiple objectives, & integration of scientific and experiential knowledge and tradition by e.g., ‘giving locals a voice’, multi-use planning & zoning	Locally-relevant & scale-appropriate conservation by e.g., nesting local MMAs in sub-district MPA	Interactive participation of diverse actors, their interests and knowledge, in conservation planning via e.g., public forums to create a more holistic understanding of marine resource needs–‘we cannot do conservation alone’	Enhanced local capacity, competency and leadership in sustainable ornamental fishery via e.g., new skills, exchange of knowledge
	Better coordinated conservation actions via cross-level, multi-stakeholder management–e.g., MPA working group, management unit	Improved local responsibility and leadership in conservation via e.g., MMA groups, community-based *Pecalang Segara*	Linked government and nongovernment actors in meaningful ways via e.g. extension agents or public forums	Empowered community-based conservation action–e.g., training in production and installation of artificial reef structures
	Improved social networks for interaction and knowledge sharing via institutions and forums–e.g., Lembongan Marine Assoc., MPA learning site			

#### Nusa Penida MPA

The Coral Triangle Center (CTC) is an environmental NGO that oversees two main programs across Indonesia. Its engagement with the Nusa Penida area began in 2008, when the CTC was still a subsidiary of US-based NGO The Nature Conservancy (it became an independent foundation in 2010). The Center believes that enhancing the capacity of conservation managers and practitioners is the path to improved ecosystem health, and its core values support building partnerships among stakeholders to find joint solutions (see also [49]). In the context of Nusa Penida, an NGO staff explained: ‘CTC’s role is to bring actors together…[and it] strongly advocates a collaborative approach’ (per. comm. 2013). Under the guidance of the CTC, Nusa Penida is being developed into an international ‘learning site’ to provide a platform for managers and practitioners, government agencies, community groups, scientists and NGOs to share knowledge and experiences about tropical conservation (see also [38]).

The NGO was acknowledged by just over half of respondents (57%) for its contributions to knowledge and information exchange. It has played a large technical advisory role and has worked closely with governments and stakeholders to lend expertise on the development of an MPA plan. As a government staff person stated (pers. comm. 2014), “*CTC works caused changes in our organization*. *We created new rules based on CTC recommendations*. *[Our agency] become more concerned on conservation because CTC give information about the importance of conservation for tourism in Nusa Penida*”. In 2012, CTC jointly facilitated the development of a marine tourism code of conduct in cooperation with the Klungkung government, Lembongan Marine Association, and Indonesian Marine Tourism Association. The code regulates diving and snorkeling activities specifically to protect manta rays and mola mola (sunfish). At the same time, CTC has held educational opportunities about e.g., marine ecology, fisheries net making, and coral reef monitoring across the islands. To support the development of local mangrove ecotourism (below), for instance, it conducted training among community members about basic mangrove ecology and restoration, and how to perform surveys to identify mangrove species (CBO member, per. comm. 2014). As a conduit for information exchange, several diving operators and local organizations noted that it was common practice to forward observational data to CTC, including that associated with wildlife sightings and illegal activities.

When asked about the influence of the CTC, respondents most frequently mentioned its role in linking stakeholders and building collaborative partnerships. The NGO has helped to connect district and provincial governments, NGOs, traditional authorities, community representatives and private sector operators via formal platforms such as the MPA Working Group and the subsequent Nusa Penida MPA Management Unit and Joint Patrol Team (formalized under district decree no. 30/2013). The nascent Management Unit is comprised of representatives from government, fishers’ association, traditional council (*Majelis Alit*), the Indonesian Navy, dive operators, NGOs and community groups to guide the overall management of the MPA. Other lesser formal platforms engaged by the CTC to connect organizations have included public consultation forums, training or skills sessions, and community-oriented education and awareness campaigns.

As of June 2014 the NGO had facilitated over sixty focus group discussions and stakeholder meetings, in part, as a way to ‘give locals a voice’ (CBO member, per. comm. 2014) to participate in broader discussions about the MPA. One outcome of this has been the development of an MPA zoning plan that incorporated the use preferences and customs (e.g., sacred territories, resource use patterns) of diverse stakeholder groups, including local peoples. The zoning system integrates scientific information with experiential knowledge and traditional practices to encompass areas for sustainable traditional fisheries, marine tourism, seaweed farming, local culture and tradition, biodiversity conservation and transport use, as well as accommodating other uses such as research and education. As well, the CTC has been credited by respondents for ‘increasing group unity’ (CBO member, per. comm. 2014) among particular sub-groups (e.g., tourism operators) in the MPA by helping to catalyze new institutions and forums for interaction. These have included the development of a Mangrove Tourism Association for local ecotourism operators in Nusa Lembongan, and the aided establishment of the locally-based Lembongan Marine Association (LMA) for private sector diving businesses. These institutions have in turn contributed to greater collaborative outcomes: "*the work the LMA has been doing is very unifying*" (tourism staff, per. comm. 2014).

However, connecting different organizations with differing interests, perspectives and knowledge has been no easy task. In describing the role of the CTC one respondent stated: “*I feel sorry for them…they are stuck between a rock and a hard place*” (tourism staff, per. comm. 2014), referring to the NGO’s position between conflicting stakeholder demands. Another respondent voiced frustration with the collaborative process, explaining that it is “*…all talk and no action…exhausting and demotivating…*” (tourism staff, per. comm. 2014). Over half of respondents surveyed observed and cited ongoing conflicts among organizations in the MPA. Constraints or barriers to building and strengthening future collaborative and knowledge-exchange relations were identified and are listed in Table 5.

**Table 5:** Responses for top constraints and barriers to establish or strengthen collaborative and knowledge-exchange relationships in Nusa Penida MPA and East Buleleng Conservation Zone.

Nusa Penida MPA	East Buleleng Conservation Zone
■ Lack of expertis	■ Availability of funding
■ Insufficient time	■ Lack of expertise
■ Incompatible organizational goals and priorities	■ Insufficient time
■ Availability of funding	■ Lack of or weak leadership
■ Political tensions and conflicts	■ Incompatible organizational goals and priorities
■ Lack of interest	■ Lack of interest
■ Lack of or weak leadership	■ Power imbalances
■ Competition and jealousy	■ Political tensions and/or conflicts between organizations
■ Language and cultural barriers	
■ Inadequate mechanisms for communication	
■ Lack of human resources	

#### East Buleleng Conservation Zone

This region hosts three bridging organizations, each with their own similar yet distinct role in coordinating the network. Hence, strong communication and coordination between all three organizations is crucial for network-level coordination.

#### Reef Check Indonesia

The environmental NGO was founded in 2005 as a chapter of a US-based Reef Check International, and operates in multiple sites across Indonesia. RC-I focuses on coral reef conservation and community well-being by promoting sustainable collaborative governance, science, and education and awareness (see also [50]). In East Buleleng, it works to empower local governments and communities, and assists in the development and planning of nested local and sub-regency marine management areas. One staff member explained, RC-I aims to “…*involve […] local communities*, *stakeholders and governments in the whole management process*. *[To] facilitate and assist collaboration of all components in the communities in the management of coastal and marine ecosystems*” (per. comm. 2014). The RC-I main office is located in Denpasar, three hours south of the Buleleng Regency, but a staff member is semi-permanently housed in the office of the Ministry of Marine Affairs and Fisheries, Buleleng.

In line with the above, the NGO was credited by just under half of respondents (42%) for improving collaboration, communication and the flow of information between different organizations in the network. RC-I has worked closely with government and other stakeholders in developing local-level and sub-district MPAs. Part of its programming has enabled the standardization of fish and coral species names, which has, as one NGO staff explained, facilitated the collection of biophysical and fisheries data both by and through RC-I across Buleleng (per. comm. 2014). In addition, RC-I has established the ‘GoBlue’ webpage as a digital node for information sharing about marine and coastal environments with wider audiences (i.e. outside the community).

According to respondents, RC-I has been influential in building capacity and contributing knowledge for community-based governance. In 2008, the NGO supported the development of three community groups for local Marine Management Areas (LMMA) in each of the villages of Bondalem, Tejakula and Penuktukan. These groups have since become platforms for collective community action, including the establishment of community-based *Pecalang Segara* (traditional guardians of the sea) to monitor for illegal activities and enforce traditional regulations. Around the same time the NGO established the Reef Check Center, an information and education center, to raise public awareness in nearby communities and schools. Numerous respondents from government and local organizations were quick to attribute changes in community mindset to its programming: “*I didn’t know about MMAs*, *about corals or fish*. *We thought to* use *resources*. *To take*. *[…] We are lucky to have big NGOs in Bali*” (CBO member, per. comm. 2015). Through these and other informal forums, RC-I has directed financial and human capital to carrying out skills and training workshops about marine ecology, coral reef and fisheries monitoring, reef restoration techniques, and the development of a marine tourism sector. It had also built local capacities via regular diver and EcoDiver certification of community members for the purposes of autonomous coral reef monitoring and the development of alternative livelihood opportunities.

#### DKP Buleleng

A regency-level government agency, the Ministry of Marine Affairs and Fisheries, Buleleng is responsible for the regulation of fisheries and other marine resources in the regency according to regency and provincial policies. One resource user described: “*they are like our fathers and mothers*, *they set the law*” (CBO member, per. comm. 2014). The agency’s mission is closely tied to enhancing the welfare and economic opportunities/growth for fisheries and coastal communities in the district. It works especially close with Reef Check Indonesia: at the time of data collection the Ministry housed a permanent staff of the NGO. Unlike all other bridging organizations in East Buleleng, DKP-B has the legal authority to make and/or enforce rules.

The respondents viewed the main contribution of DKP-B as enabling better collaboration about marine (regulatory) issues. At the time of data collection the government agency was hosting regular meetings with multiple stakeholders, both with villages individually and the sub-district as a whole, to share information and participate in discussions related to the establishment of a sub-district MPA and its zoning plan, including regulations about marine resource use. A staff from DKP-B was careful to point out, “*we cannot do conservation alone*” (per. comm. 2014), listing examples of MPA failures from other regions of Indonesia. Numerous respondents from local organizations and NGOs remarked on DKP Buleleng’s role in coordinating stakeholders and their interests associated with the MPA via public forums: “*…the government accommodates issues from […] organizations by organizing public consultancy*. *There are so many organizations involved*: *the NGOs*, *tourism actors*, *the fishermen groups and others [that] come to that occasion delivering their interests*, *ideas or aspirations*” (NGO staff, per. comm. 2014). As part of its regulatory programing, DKP-B employs ‘extension agents’ who are responsible for building relationships with, and regulating, local fisher associations in each of the villages, as well as carrying out related programming in the sub-districts.

In addition, numerous respondents cited the government agency for its financial and in-kind contributions to resource management and conservation initiatives in the region. One resource user put forward as example the DKP-B’s financial donation to the making of fish domes, noting ‘we do project-base work with DKP. We do not have an ongoing partnership’ (CBO member, per. comm. 2014).

The Indonesian Nature Foundation (LINI): The NGO was established in 2008 to promote community-based marine conservation and sustainable fisheries in Indonesia. LINI aims to support ecosystem conservation and restoration through science, education and capacity building with communities and local governments (see [51]). One staff member explained, ‘you cannot force people to protect the environment. You have to start by helping them with livelihoods and understanding’ (NGO member, per. comm. 2013). In East Buleleng, LINI operates largely at the community level and at present works most closely with the villages of Les and Penuktukan to foster a sustainable marine ornamental fishery.

LINI was also acknowledged by over half of respondents for its efforts in facilitating collaborations (identified by 68% of respondents) and improving information sharing (identified by 59% of respondents). In addition to introducing new knowledge and ideas via programming and training opportunities as mentioned below, the NGO has played a strong role in both the collection and management of data related to fisheries and ornamental fisheries. Using local middlemen LINI has facilitated data collection about fishers, fish species, catch numbers, fish distribution, and fish supply chains, as well as fish rearing/aquaculture data. It serves as a conduit to move information from local to high-levels, and LINI has worked closely with government on the management of fisheries data to inform future allowable catch quotas.

According to the respondents, the main contributions of LINI included capacity building via the transference of new skills and the exchange of knowledge. Importantly, both contributions were closely tied to ornamental fisheries programming. LINI has been heavily involved in transitioning ornamental fisheries practices in the area from cyanide-based to net-based and other friendly catch methods (see [41] for historical overview). The NGO has been a leader in the training of local community members in the production and installation of various types of artificial reef structures (fish domes, shrimp pots, ‘roti buaya’) both locally and across Buleleng. As of January 2014, over 100 fish domes and 1000 shrimp pots had been installed on the reef (CBO member, per. comm. 2014). The NGO has also carried out training about new practices and methods for sea and land-based aquaculture, and as of early 2015 the construction of a new facility for long-term training and research of the marine aquarium trade in Les was near completion.

## Discussion: Bridging Organizations for Governance and Conservation Outcomes

The evidence presented here indicates that bridging organizations contribute to more adaptive and collaborative forms of governance among different sets of actors and across scales and levels. This in turn drives successful conservation outcomes in Bali, with applications for the CT region and beyond. Four insights for conservation governance and the presence of bridging organizations are offered here. We draw on both quantitative SNA and qualitative data, including specific examples from the field, as a basis for each insight. Our intent here is it to draw out the opportunities and challenges associated with the inclusion of bridging organizations in facilitating adaptive governance processes that can lead to better marine conservation outcomes.

### Bridging Organizations Help to Navigate Complex and Dynamic Conservation Settings across Boundaries

Results from network analysis show that bridging organizations connect an immense diversity of organizational types spanning geographic and jurisdictional boundaries, from different sectors (e.g., fisheries, aquaculture, government, enforcement, etc.), and representing differing perspectives, knowledge and values. In East Buleleng, all three bridging organizations had established connections to government agencies, NGOs, community groups, monitoring and enforcement agencies, private sector representatives and funding organizations operating from local to international levels, and had also connected lesser/newly formalized institutions such as ornamental fishers’ associations, community marine management groups and *Pecalang Segara* (guardians of the sea). In Nusa Penida we see a similar diversity of connections by the CTC from local to international levels, including also those to the *Majelis Alit* traditional council and fishers’ and seaweed farmers’ associations. Linking across boundaries–jurisdictional, geographic, cultural–is an important consideration to achieving successful conservation outcomes in general [52–53] and in the CT region specifically (see [1,3,54])

Bridging organizations here facilitate the creation of social networks for diverse stakeholder participation in conservation. An important contribution in our cases has been the connection of local organizations to various external ones as a means to inform and engage communities in conservation (a challenge that is ongoing in the CT region–see e.g., [3,6,21]). Linking to the CTC, RC-I, DKP-B and/or LINI is the main conduit through which community-level actors are able to connect with higher-level organizations, although exceptions do exist. For instance, CTC works directly with district and provincial level governments on MPA demarcation and management, but has its largest number of connections is with local-level organizations such as private enterprises and community groups. Similarly, LINI plays a key role in connecting actors at the community level that are associated with fisheries and the marine aquarium trade, but it also connects to district governments and national/internationals NGOs.

The connection of local and higher-level conservation activities (in keeping with e.g., [1,19,20,54]) reflects another critical contribution of bridging organizations. For example, strategic linking between organizations in East Buleleng resulted in existing locally managed marine areas (MMA) providing the foundation for ‘scaling up’ to the sub-district level. The subsequent demarcation of the East Buleleng sub-district MPA is comprised of nested MMAs (though zoning is not yet finalized). In conservation, bridging organizations may be the only pathway for local voices, knowledge and interests to be represented at other scales. Increased connectivity between organizations both supports and facilitates conservation outcomes that better reflect the diversity inherent in societies, and is a first step in bridging the gap between local and regional conservation actions in the CT (e.g., [1,3,20]).

Despite the diversity of organizations bridged in each conservation network there are still issues of representation and inclusiveness of participation. In Nusa Penida, for example, snorkeling operators have been largely overlooked even though they are active users of MPA waters and, according to some, a source of conflict given a lack of regulations and enforcement: “…*snorkelers are a nightmare at the moment*” (tourism staff, per. comm. 2014). In East Buleleng, less organized groups such as those earning income from local dolphin and fishing tours were absent from MPA planning meetings. There are also likely to be additional stakeholders that emerge from the establishment of both MPAs. Without inclusion and meaningful participation of relevant stakeholders, at all relevant levels, differing and possibly conflicting views and priorities about conservation may be overlooked or ignored (see CT examples [5,34,55]). The result can undermine local acceptance, instigate resistance and, ultimately, result in conservation implementation failure. Bridging organization staff in the CT then must be cognizant of the diverse and shifting nature of stakeholders and their priorities in these settings, and provide a platform or mechanism to allow for trade-off negotiations and conflict resolution.

Unsurprisingly, linking across scales and sectors is not enough. High organizational diversity means there are also multiple and possibly competing interests or agendas. Decisions about conservation in the CT require bridging organizations to deal with the reality of trade-offs [56,57]. This is especially important in the CTI-CFF context to identify the extent to which diverse objectives such as sustainable development, poverty reduction, food security and biodiversity reduction are mutually exclusive (sensu [3,5,34). For instance, staff from LINI expressed concerns over the perceived compatibility of ornamental fisheries and biodiversity conservation, making note: “*…we need to move away from pushing people out of conservation areas*” (per. comm. 2013). We observed some positive evidence of trade-off deliberation in the CTC-led formation of a multi-use zoning plan for the Nusa Penida MPA. As explained above, deliberation among different groups facilitated the amalgamation of objectives for fisheries, seaweed farming, marine tourism, culture and biodiversity conservation. Although still in the early stages of zoning and planning, we see similar prioritization of multi-use by DKP-B and RC-I in the East Buleleng Conservation Zone.

### The Structure and Function of a Bridging Organization Influences the Marine Governance Process

In Bali, bridging organizations come in many shapes and sizes, and thus have differing implications or outcomes for governance and conservation outcomes. In Nusa Penida, the CTC was the sole bridging organization and the most highly connected for collaboration and knowledge-exchange. No other organization came close to its central position. This means that critical relationships among organizations in the network are to a large extent created and maintained by the CTC, which allows for ease of communication and flow of information and resources (see [18] for comparison). However, high singularity also means that the Nusa Penida MPA network may be vulnerable to fragmentation should the CTC be removed or become dysfunctional. The same can be said for its centrality in the success of conservation outcomes. In contrast, three organizations shared central positions in the East Buleleng network. Their roles in Buleleng are nested and somewhat redundant, since there are examples of how each bridging organization connects slightly different sets of organizations around different issues. Partial redundancy in bridging organizations is beneficial to provide contingency and buffer in support of conservation (as per [4,6]). All three bridging organizations in East Buleleng are currently connected. However, coordination in the network as a whole, and thus the success of conservation efforts, is still highly dependent on how and if these overlapping organizations choose to interact in the future. This reinforces the importance of knowledge-exchange platforms, addressed in the following section.

Research findings show that a bridging organization’s influence on the structure of a network (i.e. who it connects and how) varies according to type of network configuration. In other words, bridging organizations were not in all cases central in equally facilitating collaborative, knowledge-exchange and funding or resource sharing relationships (evidenced by betweenness scores). For example, LINI exhibited high importance to moderate information flow and knowledge aggregation, and much lesser importance in brokering collaborations and affecting the flow of funding and resources. On the other hand, the CTC exhibited its highest importance in brokering collaborations, and DKP-B in the flow of funding and resources. These findings imply that bridging organizations have different strengths or niches with regards to governance. Recognizing this variation is important to understand how certain bridging organizations can be engaged in the CT to achieve desired processes (e.g., sharing information, coordinating governments, improving enforcement, generating resources), and, hence, desired conservation outcomes.

Differences between bridging organizations were also found with regard to functionality. All four organizations supported some version of conservation, yet their mandates varied in priority between biodiversity conservation, livelihood development and fisheries management. Disparity between motivations or framings of conservation by an organization with high betweenness can result in differences in, for example, governance structure, scale of intervention, political processes and/or funding priorities (see e.g., [58]). This is illustrated in the case of LINI where its organizational emphasis on a community-based marine ornamental fishery has prioritized programming implemented at the local level that is focused on development of the marine aquarium trade. We suggest that the failure to recognize differences in the motivations, incentives and objectives of bridging organizations can overlook their far-reaching implications for how they shape social networks and prioritize conservation outcomes (cf. [5]).

Importantly, bridging organizations do not necessarily represent the interests or views of everyone, or do so equally. In particular, regulating who is and who is not “bridged” has implications for inclusiveness and meaningfulness of participation in conservation, as well as power (re)distribution. This in turn can influence the legitimacy and local acceptance of conservation (e.g., [4,5). It is not surprising that three of the four bridging organizations in our sites are NGOs. Not only in Indonesia, but also elsewhere in the CT, environmental NGOs play a profoundly influential role [4,59]. However, [34] notes that the proliferation of international environmental NGOs in the CT has skewed focus toward biodiversity conservation over development. When we consider formal authority in the bridging organizations in our cases, only the government agency DKP-Buleleng has official power vis-à-vis the state. The NGO bridging organizations, instead, amass power from being embedded and very central in the network. Although the influence of both types of bridging organizations is based on very different grounds and may be useful for different purposes, both are equally important to achieving successful conservation outcomes.

### ‘Bridging’ Is Accomplished Using Different Strategies and Platforms for Collaboration and Social Learning

Results from semi-structured interviews show that bridging organizations foster opportunities for community members, policy makers and practitioners to interact and share knowledge, as well as help to combine traditional, scientific and management knowledge associated with conservation–a guiding principle of the CTI-CFF [32]. [17] observe that cooperation and learning is more likely to occur among those stakeholders within a shared social network (i.e. where connections have been established).

Collaboration in this context involves the shared actions or interactions of individuals or groups, including communities, toward a collective process of decision-making [53]. In both case sites bridging organization(s) generated new avenues for face-to-face interaction through a variety of formal and informal platforms. These included mandated MPA working groups (by the CTC and DKP-B), coral reef and fisheries monitoring groups (by RC-I and LINI), and sector-specific associations (as in the case of the CTC with mangrove tourism). Such platforms have better enabled organizations to engage more directly with other agencies and identify new partners. In the case of East Buleleng, for instance, intervention by RC-I and LINI have fostered new partnerships between community groups and neighbouring hotels over their mutual interest in the development of sustainable dive tourism. In Nusa Penida, the CTC-guided nascent MPA Management Unit forms a coordinating body to represent the diverse stakeholders of the park, identify shared problems and opportunities, and work together to address undesirable social-ecological changes. Research has demonstrated that decisions generated through collaborative processes are more likely to garner broader support (see [53] for advantages and disadvantages). Importantly, however, collaboration as a normative process for conservation is likely to require a decision-making framework to allow for trade-off negotiations and conflict resolution, as mentioned above.

Social learning involves a process of iterative reflection where different actors share ideas and experiences with one another with the intent to foster collective understanding of a problem, debate solutions, and foster changes in understanding the go beyond the individual and challenge existing assumptions and practices [23,60]. Knowledge-exchange platforms catalyzed by bridging organizations in our case sites ranged from those at the community level, such as the East Buleleng community groups for MMAs or the Lembongan Marine Association for diving businesses in Nusa Penida, to the international level, as in the case of the CTC-led designation of Nusa Penida MPA as an international learning site for practitioners across the CT and beyond. These platforms and networks provide opportunity for peer-learning across vertical (local, regency, province and so on) and/or horizontal (local to local organizations) scales by serving as arenas for the experiences, objections, perspectives, and information of various organizations to be exchanged, negotiated and synthesized. We see some evidence of the responses or outcomes of social learning for conservation in our sites. For example, a series of community and stakeholder meetings organized by the CTC served as common ground to share and integrate scientific information with the experiential knowledge and tradition of local groups in the design of a multi-use zoning plan in Nusa Penida that incorporated biodiversity, livelihood and cultural factors.

Learning also involves the development of requisite knowledge and skills to engage in conservation. Research findings highlight the importance of bridging organizations to introduce and transmit information and knowledge among network members. In East Buleleng, for instance, RC-I founded an educational facility that spread awareness and understanding of marine conservation issues to surrounding communities. Similarly, partnerships with the CTC have provided government agencies with access to technical knowledge and expertise about MPA planning. In yet another example, LINI has worked with regency government to train staff on the management of fisheries data to inform future total allowable catch quotas. Training of community leaders, skills workshops and educational programming were also among the strategies used to build community capacity to engage in conservation. RC-I has trained local community members in East Buleleng to carry out autonomous coral reef monitoring on their behalf, while the CTC organizes an annual reef-monitoring program in Nusa Penida carried out alongside local diving businesses. Each bridging organization in our study sites provided a forum(s) and incentive(s) to foster broad collaboration, knowledge-exchange and learning to better align conservation outcomes with the heterogeneous social, cultural, economic and political realities of the CT context (as per [6,21]).

### Bridging Organizations Enhance Flexibility for Marine Governance and Conservation

Situated in the unique position at the nexus of where organizations meet and information and knowledge flow, a bridging organization is a space where holistic understanding might be developed and opportunities for innovation shared. Proficiency of the many different organizations in a network means that staff within bridging organizations will tend to know who to connect, how to connect them, and when. For instance, in 2013 CTC staff guided a collective response to damage caused by the installation of new underwater electrical cables between mainland Bali and the Nusa Penida islands. It engaged key local experts trained in scientific diving and monitoring to survey the damage, and coordinated a response among local communities, the Lembongan Marine Association and others, which it communicated to the electricity company PT PLN in charge of the installation. In response, the company agreed to support reef rehabilitation efforts in the area (although the program has yet to be started) [61]. The flexibility to draw upon appropriate actors and resources (e.g., experts, funding sources, information holders) allows for more coordinated and timely responses to conservation challenges in times of crisis. It also increases the impact or extent of conservation outcomes compared to responses or action undertaken by a single individual/organization alone by drawing on the collective knowledge and resources of the many.

The structure of some bridging organizations themselves is flexible. Compared to the government bridging organization, all three NGO bridging organization in this paper exhibited relatively high flexibility. None subscribed to fixed structures with regular, regimented programming that would require a large administration, and all three utilized community members as organizational extensions (to carry out e.g., data collection, programming, facilitating). For example, the bridging organization LINI employed local community members to act as ‘middlemen’ to collect data on fish catch and fish supply chains in surrounding communities. This flexible structure can also accommodate new actors and interests as they emerge, shifts to address more pressing conservation demands, and takes advantage of opportunities as they arise (e.g., funding, partnerships, networks). In addition, flexibility in bridging organization structure allows it to shift its role/programming depending on current need. For instance, two of the bridging organizations described in this paper identified their current function as ‘filling the gaps’ in conservation and management by e.g., building capacity, coordinating stakeholders. Yet, both organizations viewed this role as temporary, and expressed future plans to shift from coordinating to advisory roles.

However, a drawback to organizational flexibility concerns the consistency and long-term viability of conservation programming. Infrequent or lack of continuity between conservation programming carried out by bridging organizations may result in non-standardized practices, gaps, redundancies, or omission of technical competency (see Indonesia example in [35]). As well, skills or knowledge gained from the delivery of one-off training, without subsequent follow-up, can be quickly lost.

## Conclusions

Conservation challenges experienced in the CT, and Bali specifically, require governance approaches that are collaborative and adaptive across scales. Conservation actions must be better coordinated, information flows improved, and knowledge better integrated [52–53]). Still, fragmentation of governance, human, technical and financial deficiencies, and the ‘messiness’ inherent in contemporary conservation settings hinder effective governance regime for successful conservation outcomes in Indonesia (see [3,4]). The bridging organizations analyzed here demonstrate that they can and do play a profound role in nurturing conservation networks, and subsequently interactive processes for adaptive marine governance. For instance, we have outlined the many ways in which these organizations have allowed cooperation and built pathways for interaction, knowledge-exchange, and resource sharing, and have served as arenas/platforms for collaboration, capacity building and learning. In addition, we highlight the diverse structures and functions of these organizations, and their unique flexibility to adjust to changing contexts and needs (although not equally so). Using examples from both case sites we illustrate how bridging organizations have implications for conservation outcomes. We document, for example, a better balancing of multiple objectives and interests, greater coordination of efforts across scales, and encompassing diverse conservation actors, empowerment and capacity building for community-based conservation and leadership.

These research findings contribute to a relatively new body of literature on bridging organizations in conservation contexts and add much needed empirical investigation. By drawing on adaptive governance and social network literatures together, we gain complementary insights on how bridging organizations shape conservation networks, and the implications of this for conservation governance. The benefits of applying SNA in a range of environmental settings are only just beginning to emerge [47,48]. In this paper, we combined quantitative SNA and qualitative methods to demonstrate how knowledge gained about bridging organizations through the analysis of networks in conservation governance could be further studied with the application of interviews, participatory observation and literature review. This mixed method approach added value to the research by allowing both an ‘outsider’ perspectives in terms of the structural positions of bridging organizations and their relational characteristics in networks, and also gain data on bridging organizations from an ‘insider’ perspective, including perceptions and conservation outcomes for those involved. Future research may benefit from the inclusion of other quantitative SNA measures, such as measures of edge centrality (cf. [62]), to provide further insight on questions such as e.g., how strong are relationships with bridging organizations? are some relationships more important than others? and how well connected are bridging organizations to one another?

Yet, the bridging organizations assessed here are relatively new and their long-term impacts are uncertain. Our findings highlight a need for additional research on the role of power, motivation, agenda setting and the policy narratives that shape conservation efforts [58]. For example, how do bridging organizations promulgate particular narratives or agendas? And what are the implications of this for the actors, actions and conservation outcomes? How do bridging organization (re)distribute power in conservation? Who is included or excluded? In this regard, [58] draw attention to how the framing of conservation challenges and opportunities in the CTI-CFF have material effects on the design and implementation of conservation initiatives and programmes in the CT.

We derived our findings from two networks in Bali. However, the insights are applicable to other conservation contexts. In the CT region, including Indonesia, coastal-marine systems encompass multiple administrative jurisdictions, cultural systems and socio-economic diversity (e.g., [1–4,33]) that call for innovative multi-level and pluralistic solutions to governance challenges. The insights from this analysis show how bridging organizations add value to heterogeneous networks in conservation settings, and the importance of these organizations to governance. Understanding what organizations occupy bridging positions and how they utilize that position is important to achieve conservation outcomes. At stake are biodiversity and ecosystems of global importance, and the wellbeing of millions of people who depend on those ecosystems.

## Supporting Information

S1 Table Top ten betweenness scores for organizations in the Nusa Penida MPA network. The ID is composed of the type of organization, and a unique number to distinguish them from others in the group. Organizations here are labeled as Coral Triangle Centre (CTC), community-based organization (CBO), non-government organization (NGO), government agency (GA), monitoring and enforcement agency (ME), traditional authority (TA), and private enterprise (Pv).
(DOCX)

S2 Table Top ten betweenness scores for organizations in the East Buleleng Conservation Zone Network. The ID is composed of the type of organization, and a unique number to distinguish them from others in the group. Organizations here are labeled as Reef Check Indonesia (RC-I), Ministry of Marine Affairs and Fisheries, Buleleng (DKP-B), Indonesia Nature Foundation (LINI), fishers’ association (Fi), ornamental fishers’ association (Fo), community-based organization (CBO), non-government organization (NGO), government agency (GA), monitoring and enforcement agency (ME), and private enterprise (Pv).
(DOCX)
