# Supplementary material for: Bridging Organizations Drive Effective Governance Outcomes for Conservation of Indonesia’s Marine Systems
Source: PLoS One. 2016 Jan 21;11(1):e0147142. doi: 10.1371/journal.pone.0147142 (PMC4721869; doi:10.1371/journal.pone.0147142)
Supplement: S1 Table — The ID is composed of the type of organization, and a unique number to distinguish them from others in the group. Organizations here are labeled as Coral Triangle Centre (CTC), community-based organization (CBO), non-government organization (NGO), government agency (GA), monitoring and enforcement agency (ME), traditional authority (TA), and private enterprise (Pv). (DOCX) [file pone.0147142.s001.docx]

**S1 Table**. **Top ten betweenness scores for organizations in the Nusa Penida MPA network.**

The ID is composed of the type of organization, and a unique number to distinguish them from others in the group. Organizations here are labeled as Coral Triangle Centre (CTC), community-based organization (CBO), non-government organization (NGO), government agency (GA), monitoring and enforcement agency (ME), traditional authority (TA), and private enterprise (Pv).

| **Collaboration** | | **Knowledge-exchange** | | **Funding or resource-sharing** | |
| --- | --- | --- | --- | --- | --- |
| **Org. ID** | **between** | **Org. ID** | **between** | **Org. ID** | **between** |
| CTC | 1158.3 | CTC | 839.3 | CTC | 491.5 |
| CBO01 | 338 | CBO02 | 430 | GA03 | 315 |
| GA03 | 331.6 | GA03 | 245 | Pv02 | 146.5 |
| NGO03 | 331.2 | CBO07 | 222.7 | CBO08 | 106.5 |
| Pv02 | 269.8 | GA01 | 197.2 | CBO01 | 46.5 |
| CBO08 | 214.1 | Pv01 | 102.3 | Pv03 | 29.5 |
| CBO06 | 198.83 | CBO08 | 75.5 | TA01 | 26 |
| CBO02 | 157.68 | Pv08 | 63 | CBO06 | 23.5 |
| CBO03 | 150.67 | NGO05 | 61.3 | CBO02 | 17.5 |
| ME01 | 149.17 | Pv02 | 49.2 | NGO06 | 6 |
