# Supplementary material for: Bridging Organizations Drive Effective Governance Outcomes for Conservation of Indonesia’s Marine Systems
Source: PLoS One. 2016 Jan 21;11(1):e0147142. doi: 10.1371/journal.pone.0147142 (PMC4721869; doi:10.1371/journal.pone.0147142)
Supplement: S2 Table — The ID is composed of the type of organization, and a unique number to distinguish them from others in the group. Organizations here are labeled as Reef Check Indonesia (RC-I), Ministry of Marine Affairs and Fisheries, Buleleng (DKP-B), Indonesia Nature Foundation (LINI), fishers’ association (Fi), ornamental fishers’ association (Fo), community-based organization (CBO), non-government organization (NGO), government agency (GA), monitoring and enforcement agency (ME), and private enterprise (Pv). (DOCX) [file pone.0147142.s002.docx]

**S2 Table. Top ten betweenness scores for organizations in the East Buleleng Conservation Zone Network.**

The ID is composed of the type of organization, and a unique number to distinguish them from others in the group. Organizations here are labeled as Reef Check Indonesia (RC-I), Ministry of Marine Affairs and Fisheries, Buleleng (DKP-B), Indonesia Nature Foundation (LINI), fishers’ association (Fi), ornamental fishers’ association (Fo), community-based organization (CBO), non-government organization (NGO), government agency (GA), monitoring and enforcement agency (ME), and private enterprise (Pv).

| **Collaboration** | | **Knowledge-exchange** | | **Funding or resource-sharing** | |
| --- | --- | --- | --- | --- | --- |
| **Org. ID** | **between** | **Org. ID** | **between** | **Org. ID** | **between** |
| RC-I | 366.5 | RC-I | 302.6 | DKP-B | 94 |
| DKP-B | 355.5 | LINI | 226.7 | RC-I | 77.2 |
| GA05 | 127.9 | DKP-B | 220.7 | CBO04 | 46.7 |
| Fo01 | 120.9 | CBO04 | 56.6 | GA06 | 28 |
| LINI | 117.5 | ME01 | 42 | CBO03 | 26 |
| GA07 | 112 | Fi05 | 34 | Pv01 | 18 |
| CBO03 | 86.8 | NGO02 | 32.7 | CBO02 | 16.7 |
| ME01 | 77.6 | CBO01 | 29 | Fo01 | 16 |
| CBO01 | 77.4 | ME02 | 27 | LINI | 10.8 |
| CBO04 | 60 | Fi02 | 23.5 | NGO01 | 6 |
